# Integrated analyses of zebrafish miRNA and mRNA expression profiles identify miR-29b and miR-223 as potential regulators of optic nerve regeneration

**DOI:** 10.1186/s12864-015-1772-1

**Published:** 2015-08-12

**Authors:** Paula I. Fuller-Carter, Kim W. Carter, Denise Anderson, Alan R. Harvey, Keith M. Giles, Jennifer Rodger

**Affiliations:** Experimental and Regenerative Neurosciences, School of Animal Biology; University of Western Australia, Crawley, 6009, WA Australia; School of Physiology, Anatomy and Human Biology, University of Western Australia, Crawley, 6009, WA Australia; McCusker Charitable Foundation Bioinformatics Centre, Telethon Kids Institute, University of Western Australia, 6008 Subiaco, WA Australia; Centre for Biostatistics, Telethon Kids Institute, University of Western Australia, 6008 Subiaco, WA Australia; Centre for Medical Research, University of Western Australia, Harry Perkins Institute of Medical Research, Nedlands, 6008 WA Australia; Current address: Ronald O. Perelman Department of Dermatology, New York University School of Medicine, New York, NY 10016 USA; West Australian Neuroscience Research Institute (WANRI), QEII Medical Centre, Verdun Street, Nedlands, 6009 Western Australia

**Keywords:** Zebrafish, microRNA, mRNA, Optic nerve, Regeneration, CNS

## Abstract

**Background:**

Unlike mammals, zebrafish have the ability to regenerate damaged parts of their central nervous system (CNS) and regain functionality of the affected area. A better understanding of the molecular mechanisms involved in zebrafish regeneration may therefore provide insight into how CNS repair might be induced in mammals. Although many studies have described differences in gene expression in zebrafish during CNS regeneration, the regulatory mechanisms underpinning the differential expression of these genes have not been examined.

**Results:**

We used microarrays to analyse and integrate the mRNA and microRNA (miRNA) expression profiles of zebrafish retina after optic nerve crush to identify potential regulatory mechanisms that underpin central nerve regeneration. Bioinformatic analysis identified 3 miRNAs and 657 mRNAs that were differentially expressed after injury. We then combined inverse correlations between our miRNA expression and mRNA expression, and integrated these findings with target predictions from TargetScan Fish to identify putative miRNA-gene target pairs. We focused on two **over-expressed** miRNAs (miR-29b and miR-223), and functionally validated seven of their predicted gene targets using RT-qPCR and luciferase assays to confirm miRNA-mRNA binding. Gene ontology analysis placed the miRNA-regulated genes (*eva1a, layna, nefmb, ina, si:ch211-51a6.2, smoc1, sb:cb252*) in key biological processes that included cell survival/apoptosis, ECM-cytoskeleton signaling, and heparan sulfate proteoglycan binding,

**Conclusion:**

Our results suggest a key role for miR-29b and miR-223 in zebrafish regeneration. The identification of miRNA regulation in a zebrafish injury model provides a framework for future studies in which to investigate not only the cellular processes required for CNS regeneration, but also how these mechanisms might be regulated to promote successful repair and return of function in the injured mammalian brain.

**Electronic supplementary material:**

The online version of this article (doi:10.1186/s12864-015-1772-1) contains supplementary material, which is available to authorized users.

## Background

The regenerative capacity of the CNS in adult mammals is limited, with minimal axonal re-growth, death of damaged neurons and long-term loss of function [1]. By contrast, fish have the remarkable ability to repair most functional components of the CNS [2, 3] making them a valuable model in which to identify key molecular mechanisms involved in neural regeneration [4–6]. Anamniotes (eg fish) and amniotes (eg mammals) have a high degree of conservation at the level of both nucleotide sequence and amino acid functional domains, resulting in similar molecular and cellular processes in neural development and function of both clades. Therefore, it is possible that genes required for successful CNS regeneration are present in both fish and mammals, but that key regulatory differences in the expression of these genes underpin the differing levels of neuronal survival and axonal regeneration in different species.

MicroRNAs (miRNAs) are an extensive subclass of regulatory non-coding RNAs that repress gene expression at a post-transcriptional level by affecting mRNA translation and stability [7].  miRNAs have been implicated in many aspects of development and homeostatic pathways, with their actions often becoming more pronounced under conditions of physiological or pathological stress [8]. miRNAs are highly abundant in the CNS, which is perhaps unsurprising given the cellular and transcriptional complexity of this tissue [9]. The high degree of conservation of miRNAs across species, combined with their ability to target multiple genes, make them a likely regulator in fundamental processes, such as the ability to regenerate neural pathways in the CNS [10, 11].

To gain further insight into the regulatory mechanisms activated following a CNS lesion, the present study sought to identify miRNA(s) that were altered in zebrafish retina after an optic nerve (ON) crush. This model provides easy access to CNS-derived retinal ganglion cells (RGCs) and their axons that connect with the **optic tectum**, thereby allowing examination of both cell survival and axonal repair. Similar tissue structure across species has allowed comparison between ON injury in a variety of vertebrate species (e.g. mammals, reptiles, amphibians, fish) as a means to investigate the molecular basis of CNS regeneration [12–15]. The most dynamic gene changes occur approximately 3–4 days post injury suggesting that this phase is a critical point that sets the scene for successful regeneration or failure [12, 13]. During this period in zebrafish, previous studies have suggested that the injured RGCs are geared towards maintaining and enhancing survival whilst simultaneously preparing for axonal outgrowth [13, 16, 17].

**Fig. 1:**
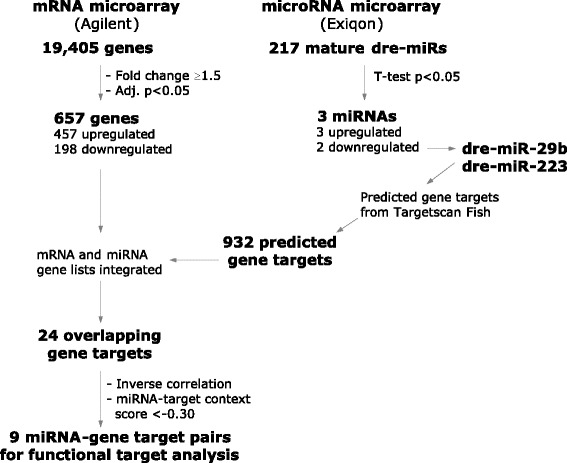
Schematic of experimental outline. Schematic flow diagram of procedures used to identify inversely correlated putative target genes of the differentially expressed miRNAs

In this study we performed a bioinformatic integration of mRNA and miRNA expression profiles of the zebrafish retina 3 days after injury, with the experimental outline represented in Fig. 1. We assessed changes in miRNA expression within the same RNA pool used for mRNA profiling as means of increasing the likelihood of finding true biological miRNA-gene relationships. The integration of these lists revealed three miRNAs that were significantly over-expressed after injury. We focused on two of these, miR-29b and miR-223, and validated seven of their target genes that were under-expressed in our dataset. Our results identified apoptotic signalling, cytoskeletal dynamics and extracellular matrix interactions as key processes activated in zebrafish following regeneration. Our data suggest that miRNAs are potential molecular targets that may be used to regulate these multiple processes in an orchestrated fashion to promote CNS regeneration.

## Results

### Injury-induced changes in gene expression and pathways

To assess the changes in gene expression after an optic nerve crush injury, we examined mRNA from whole zebrafish retinae 3 days after injury. Using an absolute log_2_-fold change cut-off of 1.5 and an adjusted *p*-value of ≤0.05, 804 transcripts were identified as differentially expressed between uninjured (control) and injured (crush) tissue and this equated to 657 genes (Additional file 1: Figure S1). Of these, 459 genes were over-expressed due to nerve injury, whilst 198 genes were under-expressed. The microarray data are available from the Gene Expression Omnibus GSE70261, http://www.ncbi.nlm.nih.gov/geo/query/acc.cgi?acc=GSE70261, and full analyses provided in the Additional Files. The top over- and under-expressed genes, ranked by a combination of *p*-value and absolute log fold change ≥1.5, are listed in Tables 1 and 2 respectively.

**Table 1:** Top upregulated genes after optic nerve crush

Ensembl ID	Gene symbol	Gene name	Fold change	adjusted *p*-value	GO process or function^a^
ENSDART00000081039	sb:cb252	sb:cb252	52.9	0.000065	Associated with mitochondria
ENSDART00000025036	gap43	Growth associated protein 43	33.9	0.000161	Tissue regeneration
ENSDART00000140944	cremb	cAMP responsive element modulator b	20.8	0.000065	DNA-dependent transcription
ENSDART00000022060	atf3	Activating transcription factor 3	13.5	0.000279	DNA-dependent transcription
ENSDART00000101970	CU571382.1	Uncharacterised protein	10.7	0.000151	
ENSDART00000126441	lepa	Leptin a	9.8	0.000161	Nervous system development
ENSDART00000110691	wnt6b	Wingless-type MMTV integration site family, member 6b	14.6	0.000690	wnt receptor signaling; neuron differentiation
ENSDART00000127420	mdp1	Magnesium-dependent phosphatase 1	6.6	0.000065	Protein tyrosine phosphatase activity
ENSDART00000105597	si:ch211-129c21.1	si:ch211-129c21.1	9.7	0.000279	Multicellular organismal development
ENSDART00000062845	mmp9	Matrix metalloproteinase 9	11.1	0.000558	Proteolysis
ENSDART00000060765	BX323876.3	Brain natriuretic peptide-like	9.7	0.000396	Inflammatory response
ENSDART00000077197	tmsb	Thymosin, beta	11.1	0.000801	Organization of cytoskeleton
ENSDART00000034377	cpa5	Carboxypeptidase A5	10.5	0.000690	Proteolysis
ENSDART00000064789	txn	Thioredoxin	5.0	0.000065	Cell redox homeostasis
ENSDART00000033494	klf6a	Kruppel-like factor 6a	4.7	0.000065	Optic nerve formation
ENSDART00000064509	stmn4l	Stathmin-like 4, like	5.8	0.000161	Regulation of microtubule (de)polymerization
ENSDART00000129989	C14HXorf65	Chromosome X open reading frame 65	5.0	0.000100	
ENSDART00000144946	adcyap1b	Adenylate cyclase activating polypeptide 1b	7.3	0.000409	Brain development
ENSDART00000020673	f3a	Coagulation factor IIIa	5.5	0.000161	Blood coagulation; integral to membrane functioning
ENSDART00000133512	fosl1b	FOS-like antigen 1b	6.6	0.000409	DNA-dependent transcription
ENSDART00000045410	thy1	Thy-1 cell surface antigen	4.8	0.000161	Organization of cytoskeleton; focal adhesion
ENSDART00000123518	tuba1	Tubulin, alpha 1	4.7	0.000161	Optic nerve formation; regulation of microtubule processes
ENSDART00000101424	nefma	Neurofilament, medium polypeptide a	4.5	0.000161	Organization of cytoskeleton
ENSDART00000121861	prph	Peripherin	10.0	0.002781	Organization of cytoskeleton; tissue regeneration
ENSDART00000082153	itga6a	Integrin, alpha 6a	5.4	0.000409	Cell adhesion membrane functioning; integrin signalling
ENSDART00000129227	b3gnt2	UDP-GlcNAc:betaGal beta-1,3-N-acetylglucosaminyltransferase 2	4.8	0.000279	Protein glycosylation
ENSDART00000141068	sox11b	SRY-box containing gene 11b	5.2	0.000381	Neuron differentiation; response to wounding

Ranked by combined –log_10_ *p*-value and ≥1.5 absolute fold change

^a^GO terms determined by ZFIN and Uniprot databases

**Table 2:** Top downregulated genes after optic nerve crush

Ensembl ID	Gene symbol	Gene name	Fold change	Adjusted *p*-value	GO process or function^a^
ENSDART00000055936	isl2b	islet2b	0.18	0.000315	DNA-dependent transcription
ENSDART00000137322	kcnip3	Kv channel interacting protein 3, calsenilin	0.29	0.000151	Potassium channel activity; neuronal apoptotic processes
ENSDART00000051693	irx4a	Iroquois homeobox protein 4a	0.15	0.002945	DNA-dependent transcription
ENSDART00000082745	emb	Embigin	0.26	0.001049	Cell adhesion; integral to membrane functioning
ENSDART00000090267	scn4ba	Sodium channel, voltage-gated, type IV, beta a	0.28	0.000848	Voltage-gated sodium channel activity
ENSDART00000052338	irx4b	Iroquois homeobox protein 4b	0.24	0.001560	DNA-dependent transcription
ENSDART00000052802	calb2b	Calbindin 2b, (calretinin)	0.26	0.001408	Calcium ion binding; neuronal excitability
ENSDART00000027398	kcna2	Potassium voltage-gated channel, Shaker-related subfamily, member 2	0.33	0.000641	Potassium ion transport; synaptic transmission
ENSDART00000067514	rbpms2a	RNA binding protein with multiple splicing 2a	0.36	0.000558	Nucleotide binding
	Unknown	Unknown	0.44	0.000407	
ENSDART00000018351	zgc:65851	Paralog of internexin neuronal intermediate filament protein, alpha	0.28	0.001732	Neurofilament cytoskeleton organization
ENSDART00000134832	rbpms2b	RNA binding protein with multiple splicing 2b	0.42	0.000690	Nucleic acid binding
ENSDART00000114765	kcnd1	Potassium voltage-gated channel, Shal-related subfamily, member 1	0.42	0.000917	Potassium ion transport; synaptic transmission
ENSDART00000113796	cacnb3b	Calcium channel, voltage-dependent, beta 3b	0.42	0.001316	Calcium ion transmembrane transport
ENSDART00000064012	ca4a	Carbonic anhydrase IV a	0.52	0.000690	Carbonate dehydratase activity; zinc ion binding
ENSDART00000113081	gpr158	G-protein coupled receptor 158	0.51	0.000947	G-protein coupled receptor activity
ENSDART00000124112	pou4f2	POU domain, class 4, transcription factor 2	0.30	0.006744	DNA-dependent transcription
ENSDART00000031167	tfap2d	Transcription factor AP-2 delta	0.43	0.001926	DNA-dependent transcription
ENSDART00000031091	vsnl1a	Visinin-like 1a	0.45	0.001678	Calcium ion binding
ENSDART00000077838	ryr3	Ryanodine receptor 3	0.51	0.001445	Calcium ion transmembrane transport
ENSDART00000098599	si:ch211-151 h10.2	Uncharacterised protein	0.44	0.002573	
ENSDART00000127084	LOC100537452	Uncharacterized protein	0.45	0.002423	
ENSDART00000084303	kcnq3	Potassium voltage-gated channel, KQT-like subfamily, member 3	0.45	0.002495	Potassium ion transport; synaptic transmission
ENSDART00000055281	kcnc3b	Potassium voltage-gated channel, Shaw-related subfamily, member 3b	0.53	0.001560	Potassium ion transport; synaptic transmission
ENSDART00000126365	smoc1	SPARC related modular calcium binding 1	0.41	0.004141	Extracellular matrix; calcium ion binding
ENSDART00000090092	ank1	Ankyrin 1, erythrocytic	0.43	0.003530	Cytoskeletal adaptor activity
ENSDART00000023562	syt2	Synaptotagmin 2	0.50	0.002264	Calcium ion binding; synaptic transmission
ENSDART00000105932	si:dkeyp-110e4.11	Uncharacterised protein	0.43	0.004012	

Ranked by combined –log_10_ *p*-value and ≥1.5 absolute fold change

^a^GO terms determined by ZFIN and Uniprot databases

**Fig. 2:**
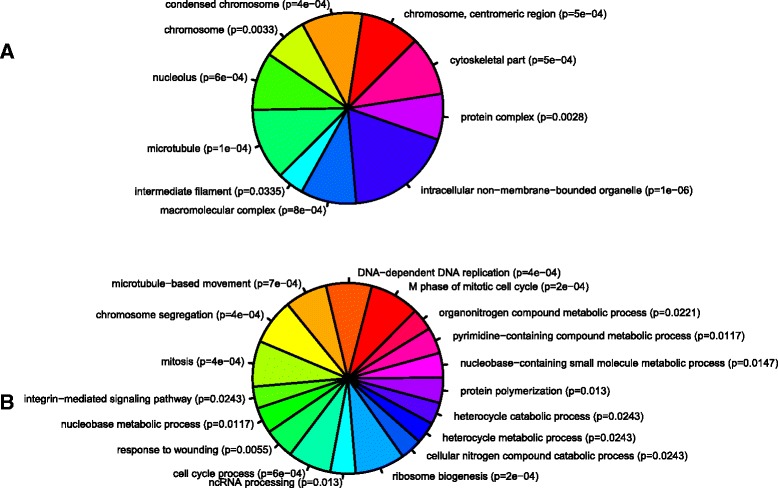
Gene ontology of over-expressed genes. GO analysis of enriched (**a**) cellular components and (**b**) biological processes of the over-expressed gene set. Each part represents -log_2_ of the *p*-value associated with the GO category from WebGestalt. WebGestalt results were filtered through GOTrim and presented only GO terms with ≥5 genes. Molecular function not shown as no terms passed this criteria.

**Fig. 3:**
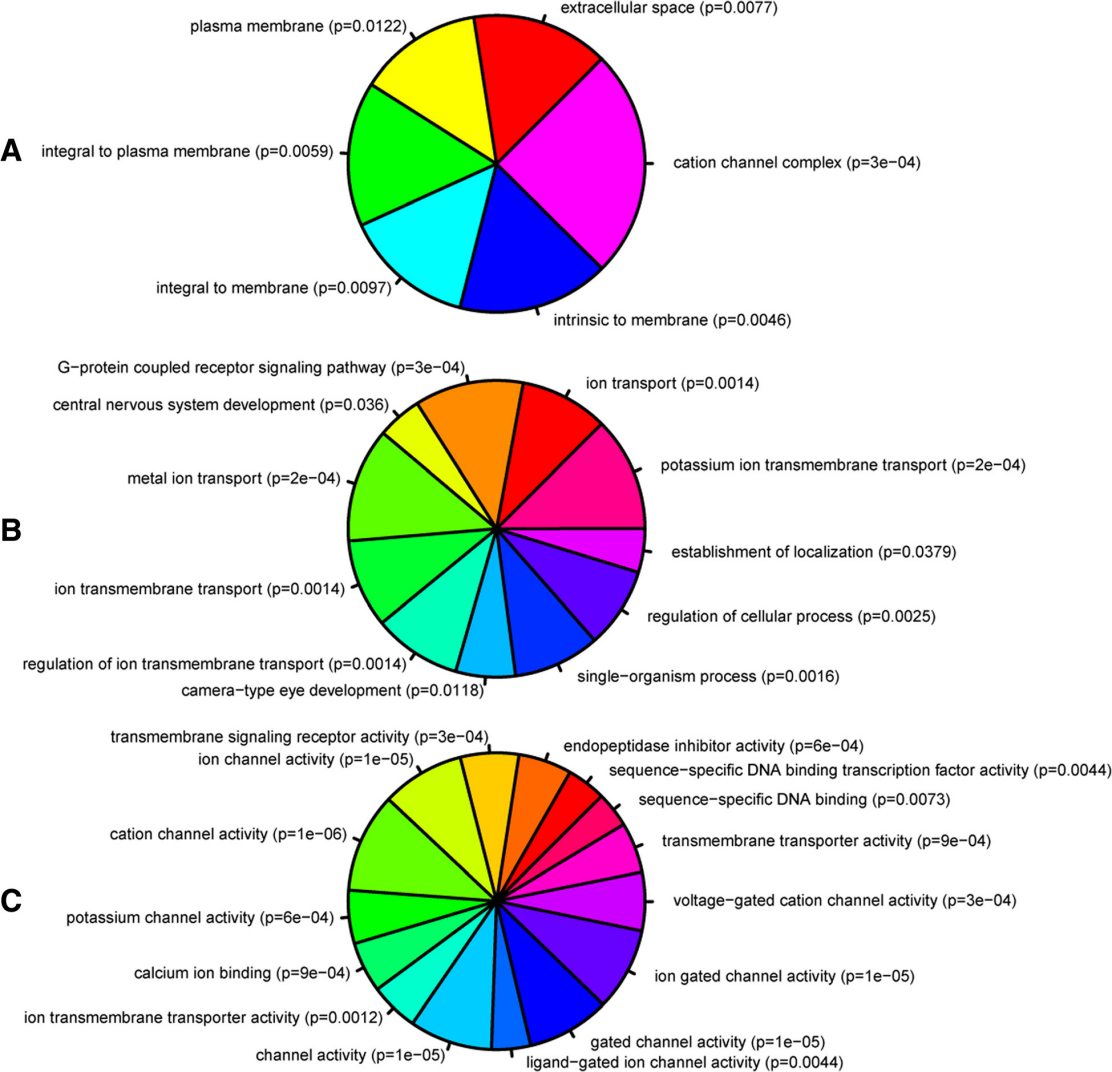
Gene ontology of under-expressed genes. GO analysis of enriched (**a**) cellular component, (**b**) biological processes, and (**c**) molecular functions of the under-expressed gene set. Each part represents -log_2_ of the *p*-value associated with the GO category from WebGestalt. WebGestalt results were filtered through GOTrim and presented only GO terms with ≥5 genes.

To determine the biological and functional implications of these expression changes by analysing over- versus under-expressed genes separately, we performed GO analysis and functional enrichment on our differentially expressed genes using WebGestalt and IPA, respectively. Figures 2 and 3 show the trimmed enriched GO terms for over- and under-expressed genes. Additional file 2: Table S1 and Additional file 3: Table S2 contain the untrimmed enriched GO terms, including IDs and *p*-values, with the full gene lists available in the Supporting data.

Enriched processes associated with over-expressed genes (Fig. 2) included ribosomal complex biogenesis, microtubule-based processes, and cell cycle activities. Other relevant processes enriched in our dataset included response to wounding (which included commonly upregulated genes, i.e. *sox11b, mmp9, prph, gap43*), and intracellular mediation of axon guidance signals (e.g. *crmp2, crmp3, crmp5a*). Our data also highlight for the first time that genes involved in non-coding RNA processing were enriched following optic nerve crush, implying that non-coding RNAs might orchestrate the regeneration process.

GO terms over-represented in the under-expressed genes (Fig. 3) revealed processes associated with eye development, DNA binding activity, G-protein coupled receptor signaling, and ion transport. In particular, transcription factors involved in eye development showed decreased expression after injury (e.g. *vax1, irx4a, pou4f2, isl2b, tbr1b, tfap2d*). The genes involved in the G-protein signaling pathways appeared to be associated with neuropeptide signaling (e.g. *tacr2, drd2a, avpr2l, pdyn, p2rx1*). Furthermore, there was a significant reduction in expression of ion transport genes, focusing on potassium and calcium channel activity.

**Fig. 4:**
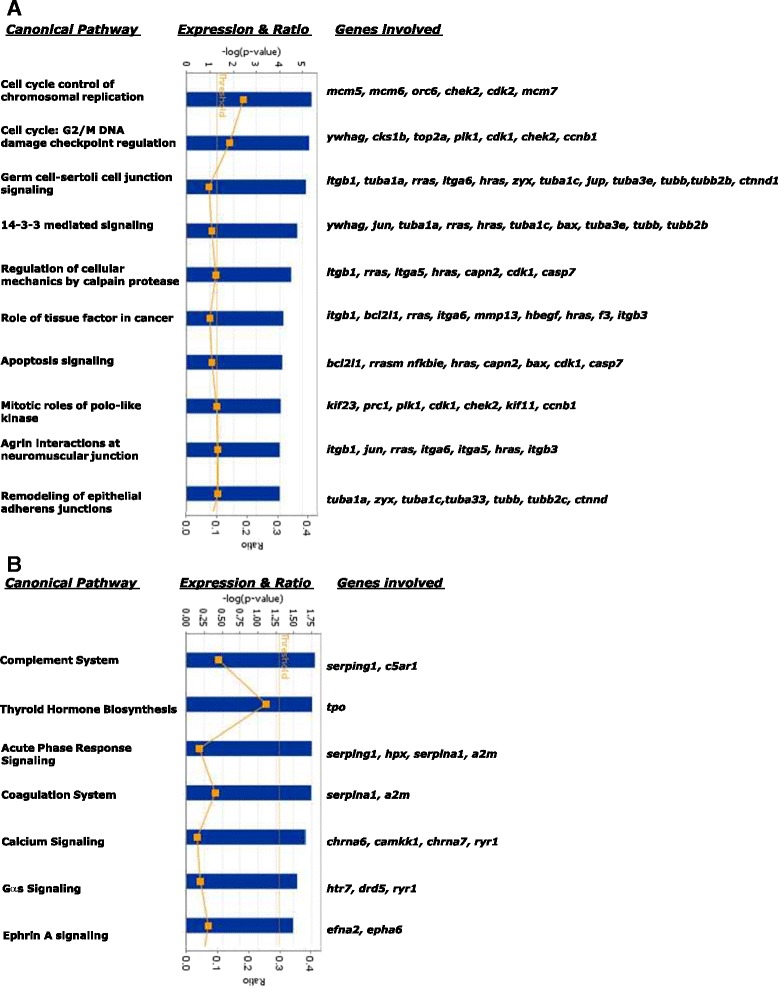
Enriched IPA canonical pathways of genes that were differentially expressed after injury. (**a**) Over-expressed and (**b**) under-expressed genes are shown

Pathway analysis of the 804 probe identifiers uploaded into IPA resulted in only a subset being used, as 481 probes were annotated but only 431 mapped to a biological function. The over-expressed genes were associated with 71 significantly enriched canonical pathways (Fig. 4a), with the top ten pathways including cell cycle regulation, 14-3-3 and apoptosis signalling, consistent with GO term results. There were fewer canonical pathways enriched in the under-expressed gene set (7 pathways), but these pathways were associated with inflammatory and immune responses, as well as calcium signaling (Fig. 4b). The majority of inflammation-associated genes were found in the acute phase response pathway (comprising *serping1, serpina1, a2m* and *hpx).* Genes specific to calcium signaling included *chrna6, chrna7, ryr1, camkk1.*

Further functional analysis revealed the relationship between genes, by placing associated genes into networks related to specific biological processes. Over-expressed genes fell into networks associated with dermatological diseases and conditions, developmental and hereditary disorders Additional file 4: Figure S2. In contrast, under-expressed genes were associated with networks involved in neurological disease, development and function of the nervous system, and organ morphology Additional file 5: Figure S3.

### Changes in miRNA expression and integration with mRNA profile identify candidate miRNA-mRNA target pairs involved in nerve regeneration

In order to examine the regulation of gene expression by miRNAs after optic nerve injury, we assessed changes in miRNA expression using the same RNA pool used for mRNA profiling. Of the 217 zebrafish specific miRNAs on the platform, we found 3 to be significantly altered 3 days after nerve crush (Table 3; all miRNA data provided in the Supporting data). We focused our subsequent investigation on two of the miRNAs over-expressed after nerve injury, miR-29b and miR-223, as their increased expression (16 and 55 % increase, respectively) is pertinent when considering the role of miRNAs is to negatively regulate their target genes [18], and unlike miR-21 [19], they have not been extensively studied in the brain.

**Table 3:** Top miRNAs differentially expressed after optic nerve

miRBase ID	miRNA name	Fold change	Adjusted *P*-value
MIMAT0001290	dre-miR-223	1.551	0.0165
MIMAT0001787	dre-miR-21	1.319	0.0361
MIMAT0001801	dre-miR-29b-2	1.159	0.0481

**Fig. 5:**
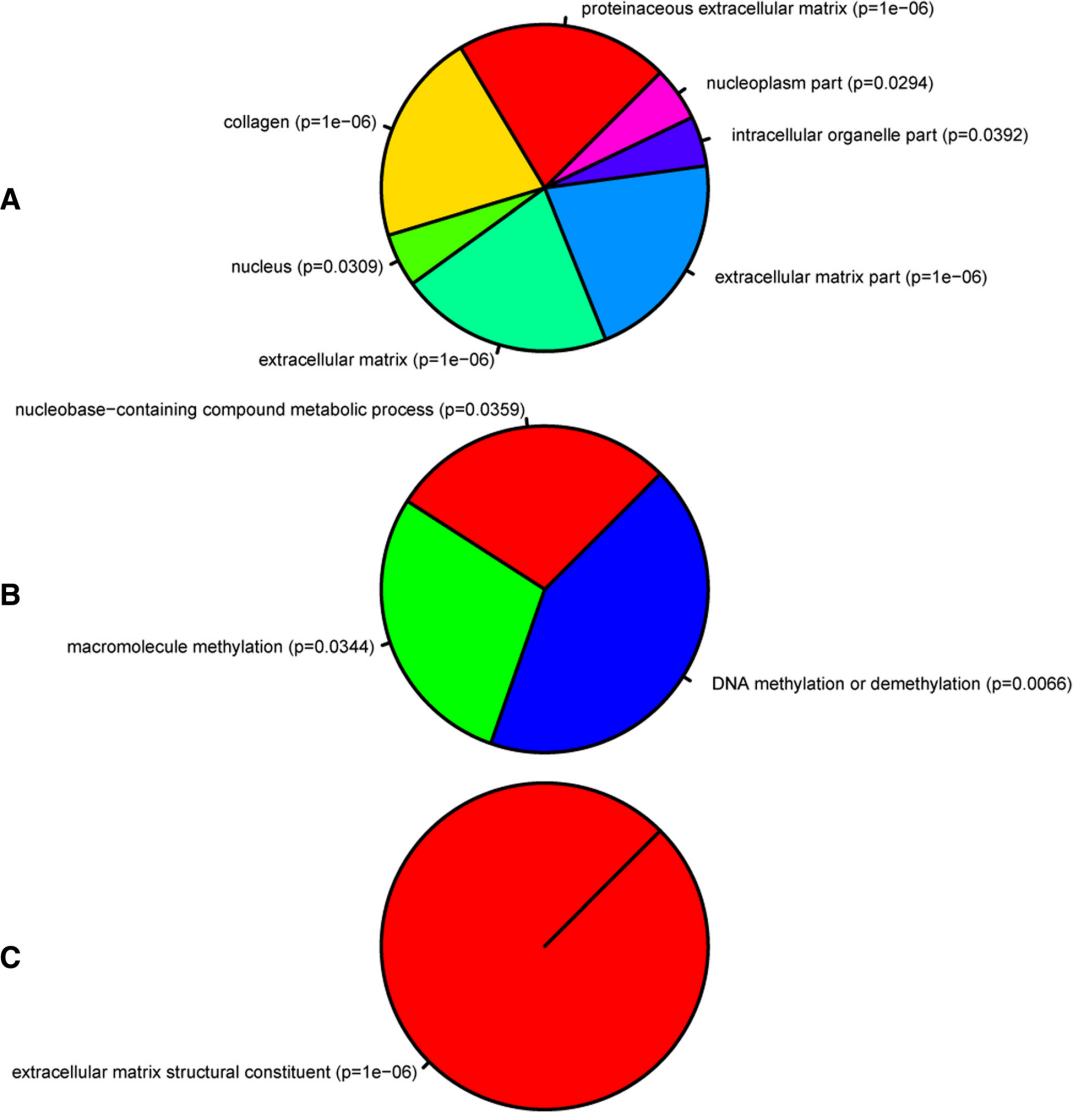
Gene ontology of miR-29b predicted targets from Targetscan Fish. Representation of GO terms associated with (**a**) cellular component, (**b**) biological process, and (**c**) molecular function. Predicted gene targets contained at least one miRNA binding site with a context score of ≤ −0.30. Each part represents -log2 of the p-value of biological process and cellular component from the set of significant biological processes and cellular components. The p-values were retrieved from gene ontology analysis in WebGestalt. A list of genes in each GO category is in Additional file 6: Table S3

**Fig. 6:**
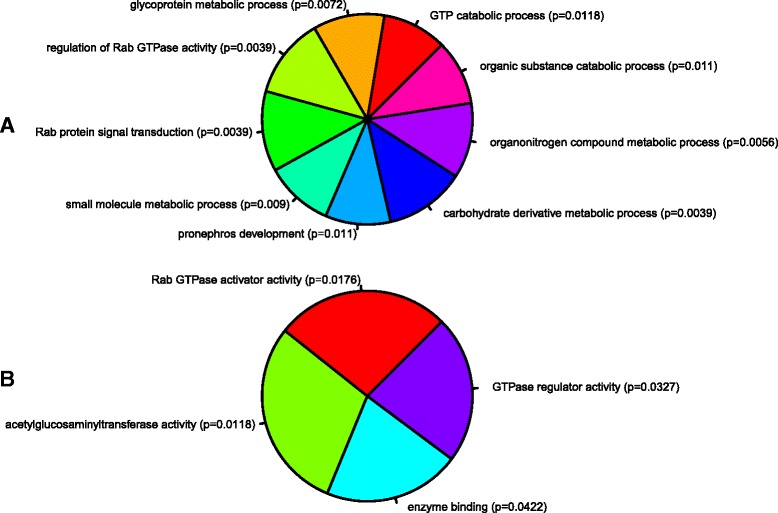
Gene ontology of miR-223 predicted targets from Targetscan Fish. Representation of GO terms associated with (**a**) biological process, and (**b**) molecular function. Predicted gene targets contained at least one miRNA binding site with a context score of ≤ −0.30. Each part represents -log2 of the p-value of biological process and cellular component from the set of significant biological processes and cellular components. The p-values were retrieved from gene ontology analysis in WebGestalt. A list of genes in each GO category is in Additional file 7: Table S4

To identify genes that are regulated by these miRNAs, we computationally identified target mRNAs that contain a putative miRNA binding site within their 3’UTR using TargetScan Fish [20], one of the few predictive databases to identify miRNA targets for species other than humans and rodents. To select gene candidates that were  most likely to be regulated by each miRNA, we used stringent parameters whereby at least one miRNA binding site had a context + score of ≤0.30. This approach resulted in 427 and 505 unique putative Ensembl target genes for miR-29b and miR-223, respectively. We then performed GO analysis on our list of predicted target genes to determine if these miRNAs where associated with any particular biological function. These results suggest a propensity for miR-29b to target genes associated with DNA modification and extracellular matrix (ECM) activities (Fig. 5, Additional file 6: Table S3), in contrast to miR-223 whose putative targets appeared to fall into nucleoside catabolic processes (particularly purine metabolism) and GTPase regulators (Fig. 6, Additional file 7: Table S4). Interestingly, glycoprotein metabolism processes (GO: 0009100) were also highlighted, with an emphasis on heparan sulfate proteoglycan (HSPG) subtypes (*p* = 0.00390). We then integrated the predicted gene list with our own differentially expressed gene set and this revealed 11 predicted target genes for miR-29b and 13 for miR-223. We refined this list by focusing on target genes whose expression was inversely correlated with the miRNA expression (i.e. potentially downregulated), and using a Targetscan context + score of < −0.30 for at least one binding site, we identified five predicted gene targets for miR-29b and four for miR-223 (Table 4).

**Table 4:** Putative miR-29b and miR-223 targets downregulated after optic nerve crush

Ensembl ID	Gene	mRNA change^a^	miRNA	Context + score^b^	No. binding sites
ENSDART00000087565	*eva1a*	0.61	miR-29b	−0.46	1 (8mer)
	eva-1 homolog a		*(miR-223)* ^*c*^	*(−0.09)*	*1 (7mer-1A)*
ENSDART00000050945	*layna*	0.45	miR-29b	−0.50	1 (8mer)
	Layilin a		*(miR-223)* ^*c*^	*(−0.16; −0.17)*	*2 (7mer-1A)*
ENSDART00000064163	*nefmb*	0.28	miR-29b	−0.41	1 (8mer)
	Neurofilament, medium polypeptide, b				
ENSDART00000018351	*ina (zgc:65851)*	0.50	miR-29b	−0.41; −0.05	2 (8mer; 7mer-1A)
	Internexin neuronal intermediate filament alpha		*(miR-223)* ^*c*^	*(−0.02; >0.03; >0.01)*	*3 (8mer; 7mer-m8; 7mer-1A)*
ENSDART00000021556	*si:ch211-51a6.2*	0.57	miR-29b	−0.33	1 (7mer-m8)
	Homolog of prss12		miR-223	−0.32; −0.07	2 (7mer-m8; 7mer-1A)
ENSDART00000126365	*smoc1*	0.41	miR-223	−0.39; > − 0.02	2 (8mer; 7mer-m8)
	SPARC related modular calcium binding 1				
ENSDART00000124670	*lrrn3*	0.65	miR-223	−0.32	1 (8mer)
	Leucine rich repeat neuronal protein 3-like				
ENSDART00000081039	^*d*^ *sb:cb252*	52.9	miR-223	−0.31	1 (7mer-m8)
	Homolog of es1-like				

^a^Fold change represents microarray expression. All genes had an adjusted *p*-value ≤0.05

^b^See Methods for summary of Targetscan Fish context + score

^c^Predicted binding sites of miR-223 in genes with a context scores less stringent then our cut-off (i.e., ≤ − 0.30)

^d^Microarray results revealed *sb:cb252* was the most upregulated gene on microarray, yet it was also one of the top predicted miR-223 target genes by Targetscan Fish; we chose to included it in subsequent experiments to determine if it was a valid miR-223 target

### Validation of putative target genes of miR-223 and miR-29b

**Fig. 7:**
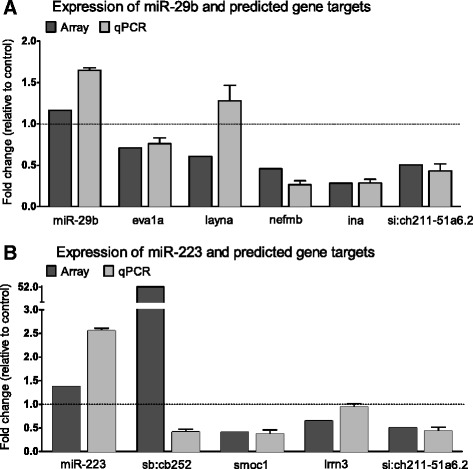
Validation of miR-29b and miR-223 putative gene targets from integration analysis. Microarray and corresponding RT-qPCR expression of genes predicted to be targeted by miR-29b (**a**) or miR-223 (**b**). The latter is presented as expression fold change (2^-ΔΔCt^) relative to PPIA (mean ± SD; *n* = 4 groups of pooled retinal RNA containing 4 animals)

**Fig. 8:**
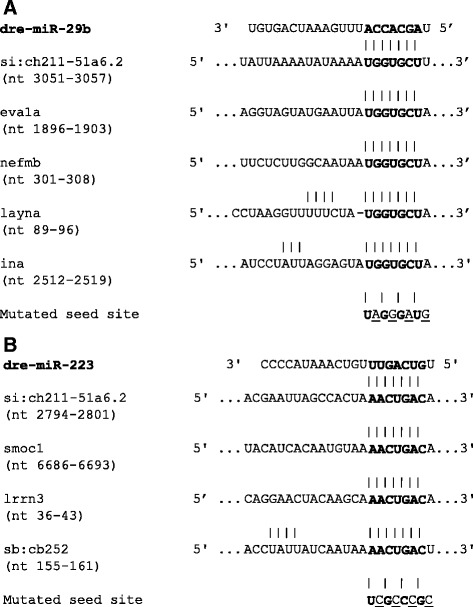
Predicted miRNA binding sites within 3’UTR of predicted target genes. Sequence of miR-29b (**a**) and miR-223 (**b**) binding sites within 3’UTR of predicted target genes (nt, nucleotide position in 3’ UTR). Seed region is bolded. Mutations predicted to disrupt miRNA-mRNA binding were made in the seed region, mutated nucleotides underlined

**Fig. 9:**
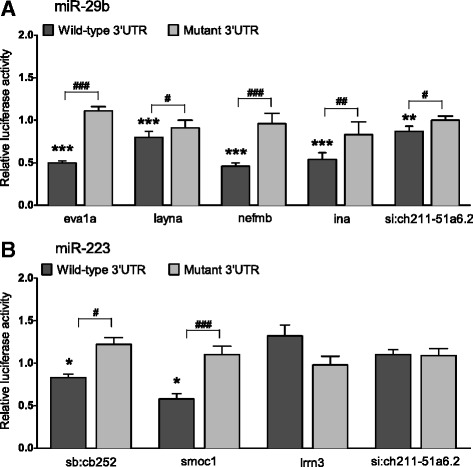
Validation of miRNA binding to 3’ UTR of putative target genes. Luciferase reporter assay of HEK293 cells cotransfected with pmirGLO plasmid containing the WT or MT 3’UTR miRNA seed sequence from each gene, and either miR-29b, miR-223 or miR-NC (scrambled control). Samples were analysed 48 h after transfection and data normalised to the pmirGLO only transfection. Columns represent the luciferase activity of either WT or MT constructs with miR-29b (**a**) or miR-223 (**b**), relative to transfection with the same construct and miR-NC. Data represents the mean ± SEM, *n* = 3 independent experiments containing 4 replicates each. Student’s *t* test comparing WT or MT construct with miRNA to miR-NC indicated as *p < 0.05, **p < 0.01, ***p < 0.001. Student’s *t* test comparing WT with miRNA to corresponding MT construct indicated as #*p* < 0.05, ## *p* < 0.01, ###*p* < 0.001

To validate our computationally predicted miRNA-gene targets, we first confirmed changes in their expression using RT-qPCR (Fig. 7), which supported our microarray expression data for all but one miRNA target, *sb:cb252.* This gene was one of the top predicted targets for miR-223 identified in Targetscan (context + score = −0.31), but our array results showed strong over-expression (53-fold). However, RT-qPCR validation revealed a decrease in expression, supporting the idea that it is negatively regulated by miR-223 as predicted by Targetscan Fish; thus the gene was included in all subsequent analyses. To functionally verify the putative miRNA-mRNA interaction, we cloned part of the 3’UTR sequence that contained the miRNA binding site of each gene into a pmirGLO vector, which contains both luciferase and renilla reporter genes (Fig. 8). A mutated version of the miRNA seed site was also created. HEK293 cells were transfected with either a wild type (WT) or mutated construct (MT), along with a miRNA mimic (miR-29/miR-223) or negative control (miR-NC). Luciferase results revealed significant inhibition of luciferase activity for miR-29b with all of its predicted gene targets (i.e., *eva1a, layna, nefmb, ina and si:ch211-51a6.2*). However, miR-223 showed statistically significant binding only to *smoc1* and *sb:cb252* (Fig. 9).

## Discussion

Here we used microarrays to examine and integrate expression of miRNA and mRNA in zebrafish retina after optic nerve crush to identify potential regulatory mechanisms involved in central nerve regeneration. Our integrated approach to studying gene regulation highlighted two miRNAs that target genes in key biological processes associated with cell survival/apoptosis, ECM-cytoskeleton signaling, and HSPG binding. Our study has provided unique information about the cellular context in which these genetic regulatory changes occur at a critical time point in the regeneration pathway.

Zebrafish are a good model in which to delineate genes associated with regeneration and contrast with mammalian studies. However, it appears there are a lot more genetic similarities between the species than first thought, with overlap between zebrafish and mammals observed in both up- and downregulated genes [13, 21–23], and even in the expression of genes associated with inhibition of regeneration (e.g., socs3 and sfpq; [24, 25]). This further supports the idea that multiple pathway analysis is required, as differences are likely to be subtle and occur at multiple points across several processes [3, 26]. It is also likely that a successful response results from the synergistic activity of several cell types, given the interaction between RGCs and other retinal cell types that are reported to assist in a positive injury response. Our approach of profiling the expression of the whole retina takes into consideration the changes that occur to in the cells adjacent to the RGCs, such as Muller glia and amacrine cells. Interestingly, the GO processes altered in our dataset overlap with several mentioned in previous zebrafish studies that have utilised other ocular tissues (isolated RGCs and whole eye; [13, 21, 27]). However there were some processes that appeared unique to this study, in particular upregulation of non-coding RNAs, which supports our original hypothesis that gene regulatory processes are key to promoting successful regeneration in zebrafish.

### The miRNAs: mir-29b and miR-223

The role of microRNAs in lower vertebrates with a known regenerative ability is gaining a lot of attention, with several recent studies identifying miRNAs associated with spinal cord repair (e.g., miR-125b in axolotl, miR-133b in zebrafish; [28, 29]) and appendage regeneration (e.g. miR-196 in axolotl tail, miR-203 in zebrafish fin; [30, 31]). Less is known about miRNA-mediated regeneration within the eye, with most focus on the role of miRNAs in Muller glia cells [32, 33]. In this study, we show for the first time that miR-29b and miR-223, are significantly over-expressed after optic nerve crush. In zebrafish, miR-29b is part of the miR-29 family that comprises three intergenic members that map to chromosome 4 (miR-29a and miR-29b-2 located within 10Kb of each other; miR-29b-2 referred to as miR-29b herein), and chromosome 6 (miR-29b-1; [34]). miR-29b has been associated with ECM remodeling, and shown to have both pro- and anti-apoptotic properties depending on the CNS injury/disease model [35–38]. The other miRNA highlighted in this study, miR-223, maps to chromosome 5 in zebrafish, and has been implicated in a wide range of pathophysiologies, including cancer, muscular dystrophy and atherosclerosis [39]. Within the CNS, it has been implicated in inflammatory processes including autoimmune disorders and mammalian CNS injury [40–42]. Our gene ontology analysis confirmed a role for miR-223 in the zebrafish immune response following injury but additionally highlighted enhanced cell survival and altered cytoskeleton/ECM response. The small number of significantly altered miRNAs found in this study may result from looking at one specific time point. On the other hand, the fact that a single miRNA is able to regulate a large number of genes might negate the need for changes in many miRNAs in order to bring about widespread biological change [10]. Further, it is likely that these two miRNAs interact with each other in a synergistic manner, as evidenced by the overlapping targeting of the same genes when less stringent cut-offs were applied during our integration analysis.

### miRNA target genes: cell survival from altered mitochondrial function

After optic nerve injury, the viability of RGCs is paramount for axonal regeneration and restoration of function. Mammalian studies have shown a high degree of cell death in the retina 2–3 days after injury, in contrast to the limited death observed in zebrafish [43, 44]. This restrained cell death may be associated with several downregulated genes in our dataset that we functionally validated for the first time as targets genes of miR-29b and miR-223, including *eva1a, layna, si:ch211-51a6.2* (a homolog of *prss12*), *smoc1* and *es1*-like homolog, *sb:cb252*.

*Eva1a* is a strongly conserved transmembrane protein localized to the lysosome and endoplasmic reticulum membrane where it is thought to function as a regulator of programmed cell death processes as well as necrosis [45–47]. Another target that may play a role in mitochondrial function (and thus potentially cell survival) is the miR-223 target, *sb:cb252*. This uncharacterized gene shares significant sequence homology with its homolog, zebrafish *es1* and human *C21orf33* [48, 49]. The exact role of both *sb:cb252* and *es1* remains unknown, however they are postulated to be involved in mitochondrial function and *es1* has been observed in zebrafish retina after a retinal lesion [50]. Downregulation of these targets may protect RGCs against cell death, consistent with the >90 % survival observed in zebrafish RGCs after crush [51].

### ECM-cell signaling and indirect cytoskeleton modification

Interaction between the cell membrane and actin cytoskeleton is essential for many cellular processes, including cell shape, adhesion, migration and signal transduction [52]. The role of ECM-cell signaling in regeneration is particularly pertinent as a means of restructuring the cellular architecture in response to injury. The role of miRNAs, including the miR-29 family, in modifying ECM-cell signaling has recently been highlighted [53]. Interestingly, three of the miRNA targets we validated are associated with this process, *layna, si:ch211-51a6.2* and *smoc1.*

Layilin (*layna*) is a transmembrane receptor that mediates hyaluronan signaling by binding to the ECM cytoskeletal linker proteins, talin, radixin and merlin [54, 55], thus destabilising the cytoskeleton [56, 57]. Downregulation of *layna* in zebrafish RGCs after crush may therefore reduce hyaluronan signaling and stabilise the cytoskeleton, promoting the early stages of axonal outgrowth. Interestingly, there were no significant changes in the expression of other hyaluronan receptors within our dataset (e.g. CD44, MyD88, TLR-4).

Another biological process highlighted in our study is heparan sulfate proteoglycan (HSPG) signalling, which is known to influence CNS regeneration [58]. miR-29b and miR-223 target genes share common functions surrounding the HSPG, agrin. The miR-29b target, *si:ch211-51a6.2,* is not characterised in zebrafish but is a homologue of the serine protease, neurotrypsin *(prss12*). Neurotrypsin cleaves agrin into two fragments which are involved in neural plasticity, axonal outgrowth and synaptogenesis (shorter C-terminal peptide; [59–61]), as well as cytoskeleton remodelling (longer N-terminal peptide: [62, 63]). Furthermore, the miR-223 target, secreted modular calcium binding 1 (*smoc1*), is a member of the SPARC (secreted protein acidic and rich in cysteine) family and binds to HSPGs (including agrin) to modulate cell adhesion [64, 65]. Although it is not clear how downregulation of their expression would impact on the regenerative process, key roles for these proteins in mediating the regenerative response in fish are supported by our finding that the agrin interactions canonical pathway is enriched with upregulated genes in our dataset. Interestingly, *smoc1* has also been implicated in Smad1-dependent bone morphogenetic protein (BMP) signaling which regulates axonal regrowth in injured dorsal root ganglion neurons [66–68]. Thus the downregulation of *smoc1* may also be part of BMP activation of pro-regenerative transcription program or manipulation of cytoskeletal dynamics at the growth cone [69].

### Direct cytoskeleton modification

In addition to indirectly reorganizing the cytoskeleton by modulating membrane signaling mechanisms, our data highlight ways in which miR-29b may directly target members of the intermediate filaments of the cytoskeleton, by regulating expression of internexin (*ina*) and neurofilament-medium homolog b (*nefmb*) [70]. The latter is one of two homologs found in zebrafish (*nefma* and *nefmb*), however *nefmb* is likely to represent the zebrafish version of *nefh,* as it shares a significant homology to human *NFH*. Although some neurofilament proteins are upregulated during successful regeneration in the mammalian peripheral nervous system, and in lower vertebrates (e.g. *prph, nefm*), the overexpression of other intermediate filaments has been associated with several neurodegenerative and CNS diseases [71–73], with specifically *nefh* and *ina* potentially being biomarkers for the likes of Amyotrophic Lateral Sclerosis and neuropsychiatric Systemic Lupus Erythematosus [74, 75]. Both have been associated with the slowing of neurofilament axonal transport, resulting in neuronal aggregates which damage the endoplasmic reticulum and mitochondria in transit, ultimately leading to cell death [75–77]. Thus their downregulation during the initial stages of axonal regeneration in our study may promote regeneration by facilitating transport of neurofilament components to required cellular areas. It is interesting to note that other microtubule and actin components of the cytoskeleton, including *b-actin, a-* and *b-tubulin, nefm* and *prph*, were upregulated after injury in our dataset. This suggests that the specific downregulation of *ina* and *nefmb* may play a significant role in zebrafish injury response.

### Zebrafish as a comparative model for miRNA role in CNS regeneration

Examining the role of miRNAs in regenerative permissive species, such as zebrafish, is an important step in delineating their role in higher order vertebrate injuries. Whilst the miRNA sequence is strongly conserved between species, this does not necessarily guarantee conservation of their function [78]. In many cases the target genes are not conserved, with the turnover of miRNA binding sites within the 3’UTR of genes thought to be a significant driver of evolutionary processes [79]. However, the two miRs examined in this study are predicted to target human and rat orthologs of the genes we validated here (Table 5), suggesting that the retention of miRNA binding sites across species may provide an opportunity to manipulate gene regulation in mammals based on our findings.

**Table 5:** Predicted miR-29b and miR-223 binding of human and rat orthologs to the validated zebrafish genes

*Zfish refseq*	*Zfish ensembl*	*Zebrafish gene*	Human ortholog (context score)^a^	Rat ortholog (context score)^a^
NM_001076587	ENSDART00000087565	*eva1a*	miR-29b (−0.21)	miR-223 (−0.32)
XM_688304	ENSDART00000050945	*layna*	miR-223 (−0.41)	miR-29b (−0.14)
				miR-223 (−0.05)
NM_001123280	ENSDART00000064163	*nefmb*	No direct ortholog	No direct ortholog
			*NEFH*: miR-223 (−0.25)	
NM_199534	ENSDART00000018351	*ina*	miR-29b (−0.17)	miR-29b (−0.15)
XM_685557	ENSDART00000021556	*si:ch211-51a6.2*	No direct ortholog	-
			*PRSS12:* miR-223 (−0.08)	
NM_001201393	ENSDART00000126365	*smoc1*	miR-223 (−0.11)	miR-223 (−0.11)
XM_003201552	ENSDART00000124670	*lrrn3*	-	-
XM_003199845	ENSDART00000081039	*sb:cb252*	No ortholog	No ortholog

^a^Context score calculated by Targetscan for human and rat predictions (Grimson et al. [91])

## Conclusion

Identifying the molecular and cellular factors associated with the successful regenerative response in zebrafish may aid in identifying therapeutic targets in the damaged mammalian CNS [6]. Our results provide a basis from which to investigate the cellular processes required for central nervous system regeneration, and further studies will examine more extensively the complete repertoire of mRNA targets of miR-29b and miR-223 (e.g., using high-throughput sequencing of RNA isolated by crosslinking immunoprecipitation (HITS-CLIP) [80]). The synergistic activities of multiple miRNAs, and the redundancy in their regulation of specific target genes and signalling pathways, provides an opportunity to reengage 3’UTR targets in order to instigate a fish-like regenerative response in the mammalian CNS [4].

## Methods

### Animals and surgery

Wild type adult zebrafish (9–12 months old, we did not distinguish between males and females), *Danio rerio,* were maintained at 27 °C on a 12 h light–dark cycle. Optic nerve crush was performed as previously described [44]. Briefly, fish were anesthetized by immersion in 0.1 % Tricaine methanesulfonate (MS222; Sigma Aldrich) in Holtfreters solution pH 7.2 [81]. The right eye was deflected forward and connective tissue cut to expose the optic nerve, which was crushed with fine forceps (Dumont n^o^5) 1 mm from the back of the eye; this procedure severs all RGC axons but leaves the nerve sheath intact as a conduit for regeneration. Three days after crush injury, fish were euthanised by overdose in MS222 and the eyes were removed. Retinae were dissected out and stored in RNAlater (Ambion) according to manufacturer’s instructions. The right retinae from intact, unoperated fish were used as controls. All procedures conformed to the NHMRC guidelines for the use of animals and were approved by the Animal Ethics Committee of the University of Western Australia.

### RNA extraction

In order to generate enough sample material to be used for microarray and miRNA arrays, retinae from 4 fish were pooled. Four biological replicates were performed for each array type (i.e., 4 microarrays for crush and control tissue, each containing pooled retinal RNA from 4 fish). Total RNA from pooled tissue was extracted using Trizol reagent (Life Technologies) according to manufacturer’s instructions, and subsequently column-purified with miRNeasy kits (Qiagen). The concentration of RNA was determined using a NanoDrop spectrophotometer (Thermo Fischer) and RNA integrity was assessed using a Bioanalyzer 2100 (Agilent).

### Microarray gene expression profiling and analysis

Microarray processing of Agilent Zebrafish 4x44K V3 GE array (one-colour labeling, hybridization and scanning) was performed by the Ramaciotti Centre for Gene Function Analysis (University of New South Wales, Australia), according to the manufacturer’s (Agilent) instructions. These arrays contain 43,603 probesets, representing 19,405 unique Unigene gene targets. Pre-processing of the array data included background correction using a normal-exponential convolution model [82] and quantile normalisation [83]. The *limma* Bioconductor package [84] was used for differential expression analysis. Probesets with an absolute fold change of ≥1.5 (log_2_ fold change 0.585) adjusted *p*-value ≤0.05 were considered as differentially expressed [85]. The microarray data are available from the Gene Expression Omnibus GSE70261, http://www.ncbi.nlm.nih.gov/geo/query/acc.cgi?acc=GSE70261.

### Gene ontology and pathway analysis

To examine the biological and functional implications of differentially expressed genes, we stratified the differentially expressed genes into those that were over-expressed or over-expressed and performed gene set enrichment analysis in both, utilising the WebGestalt suite for gene ontology terms [86], and Ingenuity Pathway Analysis software (IPA), for canonical pathways and gene network analysis (http://www.ingenuity.com). For the purposes of quickly identifying key non-redundant GO terms, we utilised the GO Trimming [87] method (soft trim, 100 %) and only plotted terms with 5 or more genes, as evidenced in Figs. 2, 3, 5 and 6. We provide the full GO terms lists in the Supporting data.

### microRNA expression profiling and analysis

Total RNA from the same sample used in microarray profiling was profiled by Exiqon Services (http://www.exiqon.com). Total RNA (~425 ng of each sample) was labeled using the miRCURY LNA microRNA Hi-Power Labeling kit with Hy3 (Exiqon) according to the manufacturer’s instructions. The Hy3-labeld samples were hybridized to the miRCURY LNA microRNA Array v.11—Other species (Exiqon), which contains probes targeting 217 mature miRNAs from *Danio rerio* miRNAs miRBase v18. The hybridization was performed according to the manufacturer’s instructions on a Tecan HS480 hybridization station (Tecan, Austria) and microarray slides were scanned using the Agilent G2565BA Microarray Scanner System (Agilent Technologies, Inc., USA). Image analysis was carried out using the ImaGene 9.0 software (BioDiscovery, Inc., USA). The data were pre-processed using the robust multi-array average expression measure [88]. One control array was removed after performing QC using the *ArrayQualityMetrics* bioconductor package [89]. As with the mRNA analysis, we used *limma* for differential expression analysis, and considered miRNA targets with an adjusted *p*-value ≤0.05 of interest.

### Platform integration

To identify putative miRNA-mRNA target pairs of interest, we integrated the mRNA and miRNA platform data, and filtered with other key datasets. Briefly, we utilised Targetscan Fish (version 6.2; [20] as the source of target prediction information, and cross-referenced the top miRNAs (by unadjusted miRNA *p*-value of ≤0.05) with mRNA array targets (by fold change >1.5 and adjusted *p*-value ≤0.05). We extracted conserved and non-conserved pairs from Targetscan Fish where the mRNA-miRNA pairs were inversely correlated (to reflect the typical miRNA-mRNA relationship), and context scores were ≤ −0.3, a generally accepted filter for prioritising higher likelihood binding sites from computational predictions. Targetscan predictions in vertebrates are ranked on the predicted efficacy of targeting as calculated using the context + scores of the binding sites, which takes into account the type of seed pairing site, miRNA-target complementarity outside the seed region., local AU content, site position within 3’UTR, target site abundance, and seed pairing stability [20, 90, 91]. We integrated Uniprot [92] and GO terms, and filtered putative target pairs by those with the most complete annotations and supporting information. We further examined our two top putative miRNAs of importance by selecting the set of genes targeted genome-wide by each miRNA, with high quality (single site) context scores ≤ −0.3, and performed GO biofunction enrichment (using Webgestalt) to identify if there were common functions targeted by each miRNA.

### RT-qPCR

Reverse transcription of mRNA used 250 ng of total RNA and was performed according to manufacturer’s instructions using GoScript reverse transcription kit with random hexamer primers (Promega). Quantitative PCR was performed in triplicate on a Rotor Gene 6000 (Qiagen) using GoTaq SYBR Green Mastermix (Promega) according to manufacturer’s instructions. Primer sequences used to confirm gene expression are provided in Additional file 8: Table S5. Data were normalized to the housekeeping gene *ppia* and relative expression was calculated using the 2^-ΔΔCt^ method [93].

Reverse transcription of miRNA used 10 ng of total RNA and was performed according to manufacturer’s instructions using the TaqMan MicroRNA Reverse Transcription kit (Applied Biosystems) and TaqMan MicroRNA Assay primers for mature miR-29b, miR-223 and U6 snRNA (Assay ID 000413, 000526, 001973, respectively; Applied Biosystems). Quantitative PCR was performed using TaqMan Universal PCR Master Mix, No AmpErase UNG (Applied Biosystems) according to the manufacturer’s instructions. Levels of miR-29 and miR-223 were normalized to U6 snRNA and relative fold change between control and crush tissue was determined using 2^-ΔΔCt^ method.

### Luciferase reporter plasmids

Oligonucleotides containing either the putative binding site of miR-29b or miR-223 from the target gene 3’ UTRs (predicted by Targetscan Fish; Additional file 9: Table S6) were annealed and ligated using *NheI* and *SalI* restriction enzymes sites downstream of the luciferase reporter gene in pmirGLO Duel Luciferase miRNA target Expression Vector (Promega). An internal *NotI* site was included in the oligonucleotide sequence for clone confirmation, and sequence and orientation was verified by DNA sequencing. Mutated binding site constructs contained 4 base substitutions within the seed site predicted to disrupt miRNA binding. Where multiple bindings sites in the 3’UTR of a miRNA was predicted, the binding site with the lowest Targetscan Fish context score was used.

### Transfection and luciferase assays

The HEK293 cell line was cultured at 37C in 5 % CO_2_ with Dulbecco’s modified Eagle’s medium supplemented with 10 % foetal bovine serum and 1 % penicillin/streptomycin. Synthetic miRNA molecules (mirVana miRNA mimics) corresponding to miR-223 (Product ID: MC10903), miR-29b (Product ID: MC10432) and a negative control miRNA (miR-NC: Product ID 4464076) were obtained from Ambion (Life Technologies). HEK293 cells were seeded at 3 × 10^4^cells/well in 96-well white plate (Greiner) and were transfected 24 h later with miRNA mimics diluted in Optimem and Lipofectamine 2000 (Life Technologies). Cells were cotransfected with 40 ng of each construct and 25nM miRNA mimic. Firefly and Renilla luciferase activities were assayed 48 h after transfection using a Dual Glo Luciferase Assay System (Promega) with a luminometer (Enspire, PerkinElmer).

### Statistical analysis

For all statistical analyses not described elsewhere, we utilised Prism software (GraphPad Software). A two-tailed Student *t*-test was used when two groups were compared.

### Availability of supporting data

Microarray datasets are available from the Gene Expression Omnibus GSE70261. Additional supporting data are available from http://dx.doi.org/10.6070/H4GH9FZ9.

## Additional files

Additional file 1: Figure S1. Volcano plot of differential gene expression in zebrafish retina after optic nerve crush. Each probe on the array is represented by a single dot, with red dots signifying the 804 differentially expressed transcripts. *P-* values are presented as –log_10_ values, expression differences presented as log_2_ fold changes. We set cut off limits at *p* < 0.05 and absolute fold change ≥1.5. By these parameters, 459 transcripts were over-expressed and 198 were under-expressed after optic nerve injury. (TIFF 64 kb) Additional file 2: Table S1. Unfiltered enriched GO terms associated with over-expressed gene set. Table contains each GO category and *p*-value. (XLS 29 kb) Additional file 3: Table S2. Unfiltered enriched GO terms associated with under-expressed gene set. Table contains each GO category and *p*-value. (XLS 33 kb) Additional file 4: Figure S2. Top gene network of over-expressed genes. Top scoring IPA gene network diagram of over-expressed gene set. The enriched networks are associated with functions involved in Dermatological Diseases and Conditions, Developmental Disorder, and Hereditary Disorders, with a network score of 49. (TIFF 561 kb) Additional file 5: Figure S3. Top gene network of under-expressed genes. IPA gene network diagram of under-expressed gene set. The enriched networks are associated with functions involved in Neurological Disease, Nervous System Development and Function, and Organ Morphology, with a network score of 45. (TIFF 730 kb) Additional file 6: Table S3. Unfiltered enriched GO terms associated with miR-29b predicted target genes sourced from Targetscan Fish database. Table contains each GO category and *p*-value. (XLS 21 kb) Additional file 7: Table S4. Unfiltered enriched GO terms associated with miR-223 predicted target genes sourced from Targetscan Fish database. Table contains each GO category and *p*-value. (XLSX 9 kb) Additional file 8: Table S5. qPCR primers for validating putative miRNA target genes. (DOCX 12 kb) Additional file 9: Table S6. Oligonucleotide sequences used in luciferase constructs. (DOCX 13 kb)

## Abbreviations

BMP: Bone morphogenetic protein
CNS: Central nervous system
DNA: Deoxyribonucleic acid
ECM: Extracellular matrix
GO: Gene ontology
GTPase: Guanosine triphosphate hydrolase
HITS-CLIP: High-throughput sequencing of RNA isolated by crosslinking immunoprecipitation
HSPG: Heparan sulfate proteoglycan
IPA: Ingenuity pathway analysis
miR-NC: Negative control miRNA construct
mRNA: Messenger ribonucleic acid
miRNA: Micro ribonucleic acid
MT: Mutated construct
NHMRC: National Health and Medical Research Council (Australia)
ON: Optic nerve
RGC: Retinal ganglion cell
RNA: Ribonucleic acid
RT-qPCR: Reverse transcription—quantitative polymerase chain reaction
SPARC: Secreted protein acidic and rich in cysteine
UTR: Untranslated region
WT: Wild type
