# Supplementary figures and images for: Integrated analyses of zebrafish miRNA and mRNA expression profiles identify miR-29b and miR-223 as potential regulators of optic nerve regeneration
Source: BMC Genomics. 2015 Aug 12;16(1):591. doi: 10.1186/s12864-015-1772-1 (PMC4534052; doi:10.1186/s12864-015-1772-1)

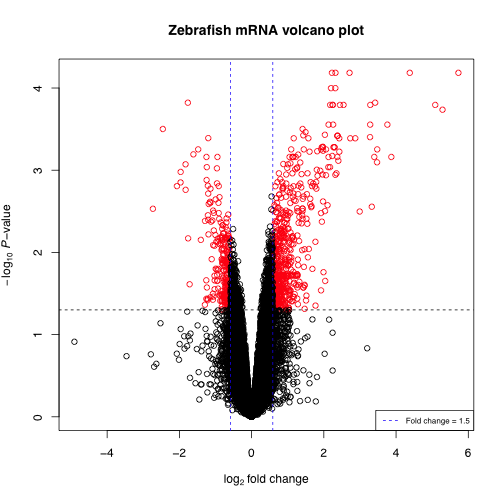

Supplement: Additional file 1: Figure S1. — Volcano plot of differential gene expression in zebrafish retina after optic nerve crush. Each probe on the array is represented by a single dot, with red dots signifying the 804 differentially expressed transcripts. P- values are presented as –log10 values, expression differences presented as log2 fold changes. We set cut off limits at p < 0.05 and absolute fold change ≥1.5. By these parameters, 459 transcripts were over-expressed and 198 were under-expressed after optic nerve injury. (TIFF 64 kb) [file 12864_2015_1772_MOESM1_ESM.tiff]

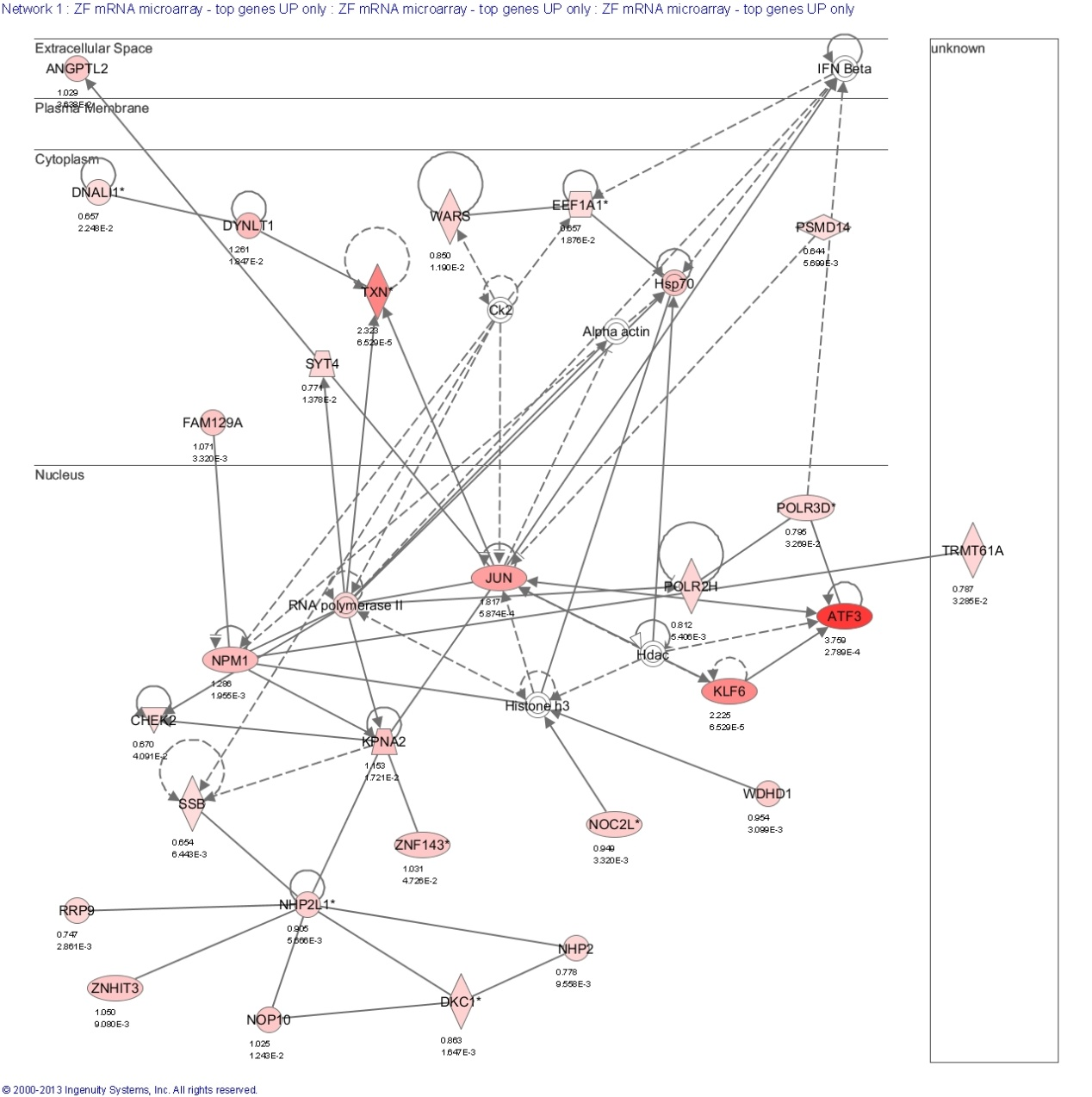

Supplement: Additional file 2: Table S1. — Unfiltered enriched GO terms associated with over-expressed gene set. Table contains each GO category and p-value. (XLS 29 kb) [file 12864_2015_1772_MOESM2_ESM.tiff]

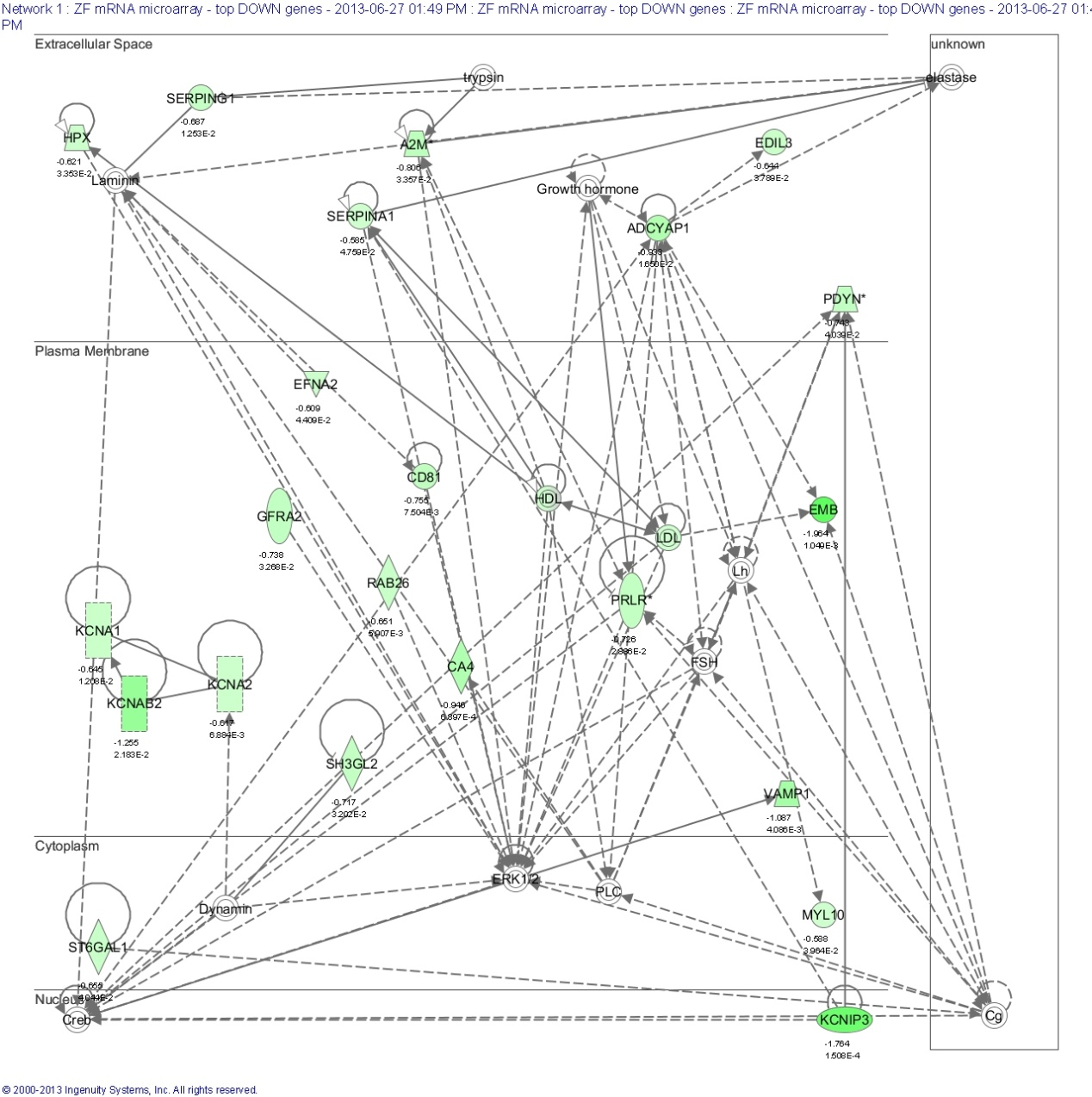

Supplement: Additional file 3: Table S2. — Unfiltered enriched GO terms associated with under-expressed gene set. Table contains each GO category and p-value. (XLS 33 kb) [file 12864_2015_1772_MOESM3_ESM.tiff]
