# Supplementary material for: Integrated analyses of zebrafish miRNA and mRNA expression profiles identify miR-29b and miR-223 as potential regulators of optic nerve regeneration
Source: BMC Genomics. 2015 Aug 12;16(1):591. doi: 10.1186/s12864-015-1772-1 (PMC4534052; doi:10.1186/s12864-015-1772-1)
Supplement: Additional file 8: Table S5. — qPCR primers for validating putative miRNA target genes. (DOCX 12 kb) [file 12864_2015_1772_MOESM8_ESM.docx]

| Additional Table 5: qPCR primers for validating putative miRNA target genes | | |
| --- | --- | --- |
|  |  |  |
| RefSeq ID | Gene symbol | Sequence (5’-3’) |
| XM_685557 | *si:ch211-51a6.2* | Forward CACTACAGAGCTCAGGGCCTC |
|  |  | Reverse TCAGACAGGAAAAGCCAGTGT |
| NM_001076587 | *eva1a* | Forward TGCATTCCGGTACCACACAA |
|  |  | Reverse CAGTAGTGGCCTGCCTTTCA |
| NM_001123280 | *nefmb* | Forward CGAAATGGGAAATGGCTCGT |
|  |  | Reverse TCCAGCAGTTTCCTGTAAGCG |
| NM_199534 | *ina (zgc:65851)* | Forward GATCGCAGCATACAGGAAGC |
|  |  | Reverse ACGTGCGACTGGTTGGATAA |
| XM_688304 | *layna* | Forward CCAAAGATAAACCTCCTGCACAC |
|  |  | Reverse GGAAGTCCAGACACAACCGT |
| NM_001201393 | *smoc1* | Forward ACCGGTATGAAAGAAGCAGGT |
|  |  | Reverse TCGTCGTTGCATTCTGGGAT |
| XM_003201552 | *lrrn3* | Forward GCTGAACCTGAGCCAGACAT |
|  |  | Reverse TGCCTTCAGGGTGCATGTAG |
| XM_003199845 | *sb:cb252* | Forward CGCTGTGGTTTTCTCTGGTTG |
|  |  | Reverse ACGGCTCAAGTGGTACATGG |
