# Supplementary material for: Integrated analyses of zebrafish miRNA and mRNA expression profiles identify miR-29b and miR-223 as potential regulators of optic nerve regeneration
Source: BMC Genomics. 2015 Aug 12;16(1):591. doi: 10.1186/s12864-015-1772-1 (PMC4534052; doi:10.1186/s12864-015-1772-1)
Supplement: Additional file 9: Table S6. — Oligonucleotide sequences used in luciferase constructs. (DOCX 13 kb) [file 12864_2015_1772_MOESM9_ESM.docx]

| Additional Table 6. Oligonucleotide sequences inserted into pmirGLO luciferase construct.  Italics show internal NotI site. Each putative miRNA binding site (as predicted by Targetscan Fish) is bolded. (S) sense primer; (AS) antisense primer. WT or MT represent wild-type or mutated binding sequences, respectively. |
| --- |

| Construct name | Oligonucleotide sequence (5’ – 3’) |
| --- | --- |
| si:ch211-51a6.2/223-WT | CTAGCTA*GCGGCCGC*T**ACGAATTAGCCACTAAACTGACA**AAACGTAAAATAGTCACGG **(S)** |
|  | TCGACCGTGACTATTTTACGTTT**TGTCAGTTTAGTGGCTAATTCGT**A*GCGGCCGC*TAG **(AS)** |
| si:ch211-51a6.2/223-MT | CTAGCTA*GCGGCCGC*T**ACGAATTAGCCACTAACCCGCCC**AAACG **(S)** |
|  | TCGACGTTT**GGGCGGGTTAGTGGCTAATTCGT**A*GCGGCCGC*TAG **(AS)** |
| lrrn3/223-WT | CTAGCTA*GCGGCCGC*TA**CAGGAACTACAAGCAAACTGACA**GAATG **(S)** |
|  | TCGACATTC**TGTCAGTTTGCTTGTAGTTCCTG**TA*GCGGCCGC*TAG **(AS)** |
| lrrn3/223-MT | CTAGCTA*GCGGCCGC*TA**CAGGAACTACAAGCAACCCGCCG**GAATG **(S)** |
|  | TCGACATTC**CGGCGGGTTGCTTGTAGTTCCTG**TA*GCGGCCGC*TAG **(AS)** |
| smoc1/223-WT | CTAGCTA*GCGGCCGC*TA**TACATCACAATGTAAAACTGACA**ACCGG **(S)** |
|  | TCGACCGGT**TGTCAGTTTTACATTGTGATGTA**TA*GCGGCCGC*TAG **(AS)** |
| smoc1/223-MT | CTAGCTA*GCGGCCGC*TA**TACATCACAATGTAAAGCCGGCC**ACCGG **(S)** |
|  | TCGACCGGT**GGCCGGCTTTACATTGTGATGTA**TA*GCGGCCGC*TAG **(AS)** |
| sb:cb252/223-WT | CTAGCTA*GCGGCCGC*TA**ACCTATTATCAATAAAACTGAC**TTGAATCTG **(S)** |
|  | TCGACAGATTCA**AGTCAGTTTTATTGATAATAGGT**TA*GCGGCCGC*TAG **(AS)** |
| sb:cb252/223-MT | CTAGCTA*GCGGCCGC*TA**ACCTATTATCAATAAACCCGCC**TTGAATCTG **(S)** |
|  | TCGACAGATTCA**AGGCGGGTTTATTGATAATAGGT**TA*GCGGCCGC*TAG **(AS)** |
| si:ch211-51a6.2/29b-WT | CTAGCTA*GCGGCCGC*TA**TATTAAAATATAAAATGGTGCTT**GATAG **(S)** |
|  | TCGACTATC**AAGCACCATTTTATATTTTAATA**TA*GCGGCCGC*TAG **(AS)** |
| si:ch211-51a6.2/29b-MT | CTAGCTA*GCGGCCGC*TA**TATTAAAATATAAAATAGGGATT**GATAG **(S)** |
|  | TCGACTATC**AATCCCTATTTTATATTTTAATA**TA*GCGGCCGC*TAG **(AS)** |
| eva1a/29b-WT | CTAGCTA*GCGGCCGC*TA**AGGTAGTATGAATTATGGTGCTA**TAGATG **(S)** |
|  | TCGACATCTA**TAGCACCATAATTCATACTACCT**TA*GCGGCCGC*TAG **(AS)** |
| eva1a/29b-MT | CTAGCTA*GCGGCCGC*TA**AGGTAGTATGAATTATAGGGATG**TAGATG **(S)** |
|  | TCGACATCTA**CATCCCTATAATTCATACTACCT**TA*GCGGCCGC*TAG **(AS)** |
| nefmb/29b-WT | CTAGCTA*GCGGCCGC*TA**TTCTCTTGGCAATAATGGTGCTA**CTTCAG **(S)** |
|  | TCGACTGAAG**TAGCACCATTATTGCCAAGAGAA**TA*GCGGCCGC*TAG **(AS)** |
| nefmb/29b-MT | CTAGCTA*GCGGCCGC*TA**TTCTCTTGGCAATAATAGGGTTG**CTTCAG **(S)** |
|  | TCGACTGAAG**CAACCCTATTATTGCCAAGAGAA**TA*GCGGCCGC*TAG **(AS)** |
| ina/29b-WT | CTAGCTA*GCGGCCGC*TA**ATCCTATTAGGAGTATGGTGCTA**ATTAG **(S)** |
|  | TCGACTAAT**TAGCACCATACTCCTAATAGGAT**TA*GCGGCCGC*TAG **(AS)** |
| ina/29b-MT | CTAGCTA*GCGGCCGC*TA**ATCCTATTAGGAGTATAGGGTTG**ATTAG **(S)** |
|  | TCGACTAAT**CAACCCTATACTCCTAATAGGAT**TA*GCGGCCGC*TAG **(AS)** |
| layna/29b-WT | CTAGCTA*GCGGCCGC*TA**CCTAAGGTTTTTCTATGGTGCTA**GATATG **(S)** |
|  | TCGACATATC**TAGCACCATAGAAAAACCTTAGG**TA*GCGGCCGC*TAG **(AS)** |
| layna/29b-MT | CTAGCTA*GCGGCCGC*TA**CCTAAGGTTTTTCTATAGGGTTG**GATATG **(S)** |
|  | TCGACATATC**CAACCCTATAGAAAAACCTTAGG**TA*GCGGCCGC*TAG **(AS)** |
|  |  |
|  | |
